# Lower Urinary Tract Dysfunction in Pelvic Gynecologic Cancer: The Role of Urodynamics

**DOI:** 10.1155/2014/303958

**Published:** 2014-11-23

**Authors:** Fouad Aoun, Alexandre Peltier, Roland van Velthoven

**Affiliations:** ^1^Jules Bordet Institute, 1 Rue Héger Bordet, 1000 Brussels, Belgium; ^2^Université Libre de Bruxelles, 50 Franklin Roosevelt Avenue, 1050 Brussels, Belgium

## Abstract

The exact incidence of lower urinary tract dysfunction is not known and its pathogenesis is not completely understood. Advances in urodynamic assessment and widespread availability of a standardized technique have facilitated its exploration prior to and subsequent to the surgical management of patients with gynecologic pelvic cancer. We performed a PubMed and Medline literature search using the following keywords: bladder dysfunction, urinary dysfunction, and urodynamics and all these terms in combination with radical hysterectomy in order to analyze the role of urodynamics in patients with pelvic gynecologic cancer in the preoperative as well as in the early and late postoperative settings.

## 1. Introduction

Gynecologic cancer confers a large burden among women worldwide. In 2015, it is estimated that 101,080 women in the United States will be diagnosed with a gynecologic cancer [1]. The incidence of gynecologic cancer will continue to increase over the next 40 years but the mortality rates will decrease perhaps due to an increase in cure rates [1, 2]. In fact, the prognosis of localized gynecologic cancer is excellent with 5-year survival rates >90% [2, 3]. Radical pelvic surgery represents the cornerstone in the management of these tumors. However, radical pelvic surgery is associated with a significant amount of complications and a negative impact on quality of life [4].

Owing to the anatomical location of female reproductive organs in the pelvis, radical treatment of gynecologic cancer has understandably been associated with urological complications [5]. Injuries of the lower urinary tract and the ureters can occur during surgery and lymph node dissection. Extensive radical gynecologic surgery can also damage pelvic nerves and vascular supply of the lower urinary tract as well as pelvic floor muscles and attachments resulting in lower urinary tract dysfunction (LUTD).

This abnormality represents the most common complication after radical pelvic surgery for gynecologic cancers. However, there is significant variation in the incidence of LUTD among different studies and limited information on the pathophysiology of LUTD after this kind of surgery. New advent in urodynamic technology, especially the advent of the multichannel recording, has led to the improvement in understanding the changes in the bladder and urethral functions that occurred after radical gynecologic surgery [6]. In this review, we give a critical analysis on the evidence for LUTD after pelvic surgery for gynecologic cancer. Emphasis will be placed on the contribution of urodynamic investigation in understanding the mechanisms that lead to such disorders and in improving short- and long-term outcomes.

We performed a PubMed and Medline literature search using the following keywords: bladder* and* radical hysterectomy and urinary dysfunction* and* radical hysterectomy. Significant results and citations were reviewed manually by the authors.

## 2. Incidence of LUTD after Radical Gynecologic Surgery

The incidence of clinically significant LUTD varies among different studies from 8 to 80% [7]. This wide range in the literature is due to variable evaluation and definition of LUTD, different operation techniques, variable follow-up time, and unstandardized urodynamic investigations. In fact, very few studies used previously validated questionnaire to assess LUTD after gynecologic cancer surgery. Some studies based their evaluation on institutional internal instruments or a simple evaluation by the surgeon in the immediate postoperative period or in the outclinic consultation [8, 9]. Some authors used observational surveys with previously validated instruments sent to patients at home and evaluating retrospectively their symptoms and their quality of life [10]. Others utilized telephone interviews with the same instruments to evaluate the incidence of LUTD [11]. Moreover, the definition of LUTD varies enormously among the studies. It includes the inability to empty the bladder, dysuria, increased frequency of urination, increased micturition urgency, nocturia, bladder sensory loss, abdominal straining on micturition, urge incontinence, and stress incontinence. Another important point relates to the use of urodynamics as an assessment tool for urinary functional outcome. Complete urodynamic data are lacking in all studies published before the standardization of urodynamic disturbances according to the International Continence Society (ICS) classification [12]. Preoperative and postoperative complete urodynamic explorations are necessary to evaluate the incidence of LUTD related to surgery because up to 80% of patients already had some degree of bladder dysfunction before surgery [13]. The incidence of LUTD is higher in all studies using urodynamics but the clinical significance of these findings is still lacking. Some studies used postvoid residual urine volume or the need of clean intermittent catheterization or straining to void as assessment tools to detect clinically significant LUTD [14, 15]. The follow-up timing seems to be the major factor influencing the wide range of incidence of LUTD reported in literature. One year is mandatory to assess the incidence of long-term LUTD after pelvic cancer surgery. Studies with follow-up >1 year revealed a low rate of LUTD with respect to those with shorter follow-up [16]. Another limitation in the published literature is the retrospective design of the studies with a variable follow-up and a small sample size. In a review by Plotti et al., only two studies out of 19 were found to be of good quality being prospective with a sample size >50 patients and a follow-up >1 year [7]. Finally, in several studies, the importance of LUTD correlates with the anatomical extent of resection and nerve preservation. A recent meta-analysis demonstrated lower incidence of LUTD following nerve sparing radical hysterectomy compared to conventional radical hysterectomy [17]. However, there are no standardized techniques for the nerve sparing approach. Urinary functional outcome varies according to the technique used and the different approaches to clear the ligament and the lymph nodes from the nerves. Laparoscopic and robotic approaches appear to facilitate the preservation of pelvic nerves by allowing a fine and precise dissection in a magnified operative field and are associated with better functional outcomes [18–20]. Unilateral nerve sparing approach is also associated with lower incidence of LUTD demonstrating that unilateral nerve injury could be partially compensated by the nervous supply from the other side [21, 22]. In addition, there are different neural injuries with different prognosis. The injury could be a temporary blockade of signal transmission without axon lesion (neurapraxia) with functional disorders resolving hours to weeks following surgery. It could be a transection of the axon with intact nerve sheets (axonotmesis) allowing regeneration at the site of the injury and distal to the injury with a nerve growth velocity varying from 0.25 mm/day to 4 mm/day. A complete transection of the nerves including their sheets is also possible with no potential to regenerate [23]. The mechanism is even more complex because injury of vascular supply to these nerves is as much important as injuries to the nerves themselves [24]. The combination of stretch and ischemia makes the nerve more vulnerable to injury. It appears that to obtain optimal functional results avoiding nerve handling in nerve sparing approach should be mandatory. Finally, nerve injury is not the only mechanism responsible for LUTD after radical hysterectomy as demonstrated by the higher incidence of LUTD following class 4 nerve-sparing radical hysterectomy. Direct surgical injury to the bladder wall, lymph stasis, interruption of the blood supply, and fibrosis of the urethra also play a role [25, 26].

Stress urinary incontinence (SUI) and voiding and/or storage dysfunction increased significantly following radical pelvic surgery. LUTD is a common finding in the early postoperative period. Its incidence has been reported between 70 and 85% of cases in the literature [27–31]. With adequate urological care, bladder dysfunction resolves spontaneously within 6 to 12 months in 70% of cases [32, 33]. Early LUTD is characterized by decreased bladder capacity, detrusor underactivity, and diminished bladder sensation which causes voiding dysfunction and may require urethral catheterization [34, 35]. Late dysfunction usually reveals voiding difficulty with abdominal straining, decreased bladder compliance, detrusor overactivity, and urinary incontinence [27, 28].

For example, the overall incidence of urodynamic LUTD after radical hysterectomy is 72%. In the early postoperative period, detrusor dysfunction with low compliance is found in 24.4% (range: 18%–73%) of cases, mixed urinary incontinence in 24.5% (range: 11%–50%), and SUI in 40% (range: 11%–81%) [7]. Studies with a follow-up >12 months after primary radical surgery reported that the abovementioned rates are reduced to 35%, 17%, and 38%, respectively, and ≥16% of women experience LUTD severe enough to seek medical care [32].

Two postoperative factors modify the natural history of the functional urinary outcome. The first is clean intermittent catheterization and the second is adjuvant radiotherapy which increases the incidence of LUTD and constitutes a confusion factor for studies with a long-term follow-up [36, 37].

## 3. Importance of Urodynamics in Radical Gynecologic Surgery

In the last years, advances in urodynamic assessment techniques, especially the advent of the multichannel recording, and widespread availability of a standardized technique have facilitated exploration of LUTD prior to and subsequent to the surgical management of patients with gynecologic pelvic cancer.

### 3.1. In the Preoperative Phase

LUTD and abnormal urodynamic findings are common in patients with gynecologic pelvic cancer prior to surgical treatment. Lin et al. showed that only 17% of patients with cervical cancer had normal urodynamic findings before radical hysterectomy [13]. The authors reported voiding LUTS in 10%, storage LUTS in 45%, and both voiding and storage LUTS in 28%. In addition, 37% had genuine stress urinary incontinence, 8% urge urinary incontinence, and 6% mixed urinary incontinence [13]. However, controversies in the literature surround the causal relationship between LUTD and pelvic gynecologic cancer. The increased rate of urodynamic abnormalities prior to surgery was not associated with the stage of the disease according to one study [6]. The authors concluded that LUTD in patients with pelvic gynecologic malignancies is not due to neurologic damage [6]. It is noteworthy to mention that gynecologic malignancies occur in a group of women at risk of LUTD. Parity, age, and menopausal status might be possible factors contributing to the increased rate of LUTD. A match pair analysis between urodynamic findings in women scheduled to radical pelvic gynecologic surgery compared to women in general population without pelvic malignancy would be of great value in understanding LUTD due to the presence of the pelvic malignancy. Urodynamic assessment before surgery is not systematically recommended for all women to be treated by radical hysterectomy. Furthermore, the evidence supporting its use in the preoperative settings in symptomatic women is lacking. However, preoperative urodynamic assessment for patients included in clinical trials is mandatory in order to understand the effect of surgery on the lower urinary tract. Comparison of preoperative and postoperative urodynamic parameters had contributed to the understanding of a part of the pathophysiology of LUTD after radical hysterectomy. In fact, more nerves are damaged during radical hysterectomy than during simple hysterectomy and the increased rate of LUTD and urodynamic abnormalities after radical hysterectomy compared to simple hysterectomy had led to the development of nerve sparing techniques [38, 39]. In a recent comparative urodynamic study, Maneschi et al. found only mild functional impairments in the postoperative period after nerve-sparing radical hysterectomy in 15 patients [40]. These results support the neurologic origin of LUTD after gynecologic radical pelvic surgery.

### 3.2. In the Early Postoperative Phase

During the early postoperative phase, bladder function is reported to be affected in 70–85% of patients with a significant reduction of bladder compliance and a significant reduction of maximal urethral closure pressure [5, 16, 41]. The pathogenesis of this condition is not completely elucidated and appears to be multifactorial.

In the past, several urodynamic studies were carried out to explain bladder dysfunctions after radical gynecologic pelvic surgery. They had defined well the changes in urodynamic parameters and their incidence. In 1949, Halter and Richter reported a cystometric demonstration of a hypertonic bladder with reduced capacity in the early postoperative period [42]. Their findings have been confirmed by a number of authors. For some authors, hypertonic bladder dysfunction was the result of an increase in myogenic tonicity of the detrusor muscle secondary to the trauma of operation and prolonged catheter drainage [43]. For others, the dysfunction was attributed to partial peripheral neurologic denervation with parasympathetic dominance, and they ascribed little significance to the role of the sympathetic innervations [44, 45]. In more recent studies combining cystometry and electromyography, the presence of normal sphincter function and the absence of a decrease in bladder pressure when treated with a parasympatholytic drug had suggested that bladder dysfunction was not a result of parasympathetic dominance [43]. The role of sympathetic system in the pathophysiology of the early postoperative dysfunction was corroborated by the studies of Forney and Low et al. [46, 47]. The authors showed that the compromise of sympathetic *β*-adrenergic function during storage phase causes consequent low bladder compliance. Furthermore, the loss of sympathetic *α*-adrenergic stimulation may have an excitatory effect on parasympathetic transmission to the detrusor muscle during urine storage and a permanent relaxation of the bladder neck and the proximal urethra, explaining the high incidence of the detrusor dyssynergia and the urinary stress incontinence after radical pelvic surgery. They concluded that sympathetic loss plays a major role in postoperative bladder changes. Standardization of urodynamic studies in the late 1990 contributed to the emergence of a new theory [48]. Bladder dysfunction may be due to the unmasking of intrinsic detrusor activity, characterized by loss of *β*-adrenergic innervations with subsequent *α*-adrenergic hyperinnervation, or due to the impact of residual sympathetic innervations [49, 50]. In fact, the significant decrease of the maximal urethral closure pressure encountered in the early postoperative period could be attributed to the damage of the pelvic plexus and pudendal nerves with loss of periurethral tone. The loss of sympathetic adrenergic stimulation may have an excitatory effect on parasympathetic transmission to the detrusor muscle during urine storage and may lead to permanent relaxation of the bladder neck and the proximal urethra. These alterations could contribute to the characterization of urinary stress incontinence and detrusor overactivity and incontinence after radical pelvic surgery. Furthermore, in a recent study, Axelsen and Petersen confirmed the crucial role of the urethral sphincter mechanism [51]. They had reported no differences in urodynamic findings between continent and incontinent women after radical hysterectomy except for an overall difference in the intraurethral pressure [51]. Hamada et al. found urodynamic correlation between the severity of the incontinence and reduction of bladder compliance [49]. With the advent of *β*-agonists, it would be interesting to test its effectiveness in patients after radical hysterectomy. Inhibition of relaxation of the detrusor muscle by a *β*-agonist in the filling phase should result in reduced bladder compliance and urinary incontinence.

Another emerging concept, based on decentralization rather than complete denervation, is gaining place due to studies on animal models. It was hypothesized that damage to the afferent nerves leads to reduced and abnormal afferent input into the central nervous system, and this could interfere with the normal function of the pontine micturition center [9, 52].

Close cooperation between the gynecologists and the urologists is an essential prerequisite of good practice for the adequate management of LUTD in the early postoperative phase. The best treatment option for bladder emptying and significant postvoid residue is clean intermittent catheterization [8]. Abdominal straining to facilitate emptying the bladder is not recommended, as it leads to increased bladder pressure, increased risk of vesicoureteral reflux, and pressure propagation to the kidneys, potentially resulting in renal damage in the long term. Voiding without abdominal pressure enhances recovery of bladder function after radical hysterectomy [36]. Adequate postoperative bladder care can help to restore bladder function within 12 months of surgery. The daily frequency of clean intermittent catheterization is dependent on the residual urine volume and occurrence of spontaneous micturition. A permanent indwelling or suprapubic catheter should be avoided as long as possible as these can lead to complications such as urinary leakage, untreatable infections, bladder stones, fibrotic bladder, and bladder carcinoma.

### 3.3. In the Late Postoperative Phase

Spontaneous recovery of bladder function is generally to be expected within 6–12 months after surgery [32, 33]. The mechanisms of spontaneous recovery are complicated. It could be attributed to plasticity reorganization which occurs at multiple levels in the central and peripheral nervous system in response to peripheral injuries [9]. In a feline model, ablation of the pelvic plexus with early widespread degeneration of intrinsic axons and muscle cells was followed after 10 weeks by a period of restitution of cholinergic axon terminals, increase in adrenergic and copeptidergic axons, and muscle cell regeneration [52]. However, persistent late changes are reported in 30 to 50% of studies [36, 37]. In routine practice, the persistence of symptoms beyond 12 months is indicative of worse functional prognosis. Urodynamic studies had played a major role in understanding the natural history of postoperative LUTD. In all studies with long-term urodynamic assessment, bladder compliance is improved and maximal urethral closure pressure is stabilized from the third postoperative month. Another important point related to the use of urodynamics as an assessment tool for surgical functional outcome is low incidence of normal detrusor function in long-term studies. Benedetti-Panici et al. reported normal detrusor function in 24% of their patients 1 year after surgery. They have found acontractile detrusor in 2% of patients, overactive detrusor in 21%, urodynamic stress incontinence in 29%, and mixed incontinence in 24% [16]. The high rate of urodynamic abnormalities in their series is due in part to their rigorous observation protocol and systematic urodynamic evaluation of all patients. In clinical practice, these abnormalities are rarely associated with severe clinical manifestations as demonstrated by the study of Pieterse et al. [53]. It seems also that cancer patients deal with urinary symptoms better than noncancer patients given the relatively high level of distress that is usually present regarding their underlying malignant disease compared to the other symptoms [16]. In contrast, improvement of clinical symptoms alone is not always synonym of improved bladder function. It could be the result of compensatory factors such as abdominal straining, substitute sensation, and voiding technique. Such patients are always susceptible to lower urinary tract decompensation and upper urinary tract deterioration especially if not diagnosed at time [54]. The role of urodynamic investigation is to detect these potentially dangerous situations by demonstrating subclinical modifications in bladder function and/or urethral closure pressure.

## 4. Conclusion

LUTD is the most common complication after pelvic gynecologic surgery and it alters significantly the quality of life of the surviving patients. The pathophysiology is complex and incompletely elucidated. Urodynamic investigations played a major role in understanding the neurologic mechanisms underlying the dysfunction. These findings had led to the modification of the procedure in order to minimize urologic functional outcomes. Urodynamics played also a major role in characterizing postoperative disorder allowing a better urologic care modifying long-term LUTD and renal deterioration. Electrophysiologic studies and video-urodynamic recordings by characterizing, in a clinical trial, neurophysiologic and anatomic parameters would certainly help elucidate the mechanisms of LUTD following pelvic gynecologic cancer surgery.

## References

[1] Wallace A. H., Havrilesky L. J., Valea F. A., Barnett J. C., Berchuck A., Myers E. R. (2010). Projecting the need for gynecologic oncologists for the next 40 years. *Obstetrics and Gynecology*.

[2] Institut de Veille Sanitaire (InVS), Institut National du Cancer (INCa) (2009). *Réseau Francim , Institut National de la Santé et de la Recherche Médicale (Inserm), Hospices Civils de Lyon (HCL)*.

[3] Arbyn M., Castellsagué X., de sanjosé S., Bruni L., Saraiya M., Bray F., Ferlay J. (2011). Worldwide burden of cervical cancer in 2008. *Annals of Oncology*.

[4] Wu J., Liu X., Hua K., Hu C., Chen X., Lu X. (2010). Effect of nerve-sparing radical hysterectomy on bladder function recovery and quality of life in patients with cervical carcinoma. *International Journal of Gynecological Cancer*.

[5] Zullo M. A., Manci N., Angioli R., Muzii L., Panici P. B. (2003). Vesical dysfunctions after radical hysterectomy for cervical cancer: a critical review. *Critical Reviews in Oncology/Hematology*.

[6] Lin H. H., Sheu B. C., Lo M. C., Huang S. C. (1998). Abnormal urodynamic findings after radical hysterectomy or pelvic irradiation for cervical cancer. *International Journal of Gynecology and Obstetrics*.

[7] Plotti F., Angioli R., Zullo M. A., Sansone M., Altavilla T., Antonelli E., Montera R., Damiani P., Benedetti Panici P. (2011). Update on urodynamic bladder dysfunctions after radical hysterectomy for cervical cancer. *Critical Reviews in Oncology/Hematology*.

[8] Chuang F.-C., Kuo H.-C. (2009). Management of lower urinary tract dysfunction after radical hysterectomy with or without radiotherapy for uterine cervical cancer. *Journal of the Formosan Medical Association*.

[9] Chen G.-D., Lin L.-Y., Wang P.-H., Lee H.-S. (2002). Urinary tract dysfunction after radical hysterectomy for cervical cancer. *Gynecologic Oncology*.

[10] Brooks R. A., Wright J. D., Powell M. A., Rader J. S., Gao F., Mutch D. G., Wall L. L. (2009). Long-term assessment of bladder and bowel dysfunction after radical hysterectomy. *Gynecologic Oncology*.

[11] Kives S., Lefebvre G. (2010). Supracervical hysterectomy. *The Society of Obstetricians and Gynaecologists of Canada Clinical Practice Guideline no.*.

[12] Abrams P., Cardozo L., Fall M., Griffiths D., Rosier P., Ulmsten U., Van Kerrebroeck P., Victor A., Wein A. (2003). The standardisation of terminology in lower urinary tract function: report from the standardisation sub-committee of the International Continence Society. *Urology*.

[13] Lin H.-H., Yu H.-J., Sheu B.-C., Huang S.-C. (2001). Importance of urodynamic study before radical hysterectomy for cervical cancer. *Gynecologic Oncology*.

[14] Querleu D., Narducci F., Poulard V., Lacaze S., Occelli B., Leblanc E., Cosson M. (2002). Modified radical vaginal hysterectomy with or without laparoscopic nerve-sparing dissection: a comparative study. *Gynecologic Oncology*.

[15] Trimbos J. B., Maas C. P., Deruiter M. C., Peters A. A. W., Kenter G. G. (2001). A nerve-sparing radical hysterectomy: guidelines and feasibility in Western patients. *International Journal of Gynecological Cancer*.

[16] Benedetti-Panici P., Zullo M. A., Plotti F., Manci N., Muzii L., Angioli R. (2004). Long-term bladder function in patients with locally advanced cervical carcinoma treated with neoadjuvant chemotherapy and type 3–4 radical hysterectomy. *Cancer*.

[17] Long Y., Yao D.-S., Pan X.-W., Ou T.-Y. (2014). Clinical efficacy and safety of nerve-sparing radical hysterectomy for cervical cancer: a systematic review and meta-analysis. *PLoS ONE*.

[18] Park N. Y., Chong G. O., Hong D. G., Cho Y. L., Park I. S., Lee Y. S. (2011). Oncologic results and surgical morbidity of laparoscopic nerve-sparing radical hysterectomy in the treatment of figo stage ib cervical cancer: long-term follow-up. *International Journal of Gynecological Cancer*.

[19] Puntambekar S. P., Patil A., Joshi S. N., Rayate N. V., Puntambekar S. S., Agarwal G. A. (2010). Preservation of autonomic nerves in laparoscopic total radical hysterectomy. *Journal of Laparoendoscopic & Advanced Surgical Techniques*.

[20] Magrina J. F., Pawlina W., Kho R. M., Magtibay P. M. (2011). Robotic nerve-sparing radical hysterectomy: feasibility and technique. *Gynecologic Oncology*.

[21] Kato T., Murakami G., Yabuki Y. (2003). A new perspective on nerve-sparing radical hysterectomy: nerve topography and over-preservation of the cardinal ligament. *Japanese Journal of Clinical Oncology*.

[22] Zhu T., Yu A. J., Shou H. F., Chen X., Zhu J. Q., Yang Z. Y., Zhang P., Gao Y. L. (2011). Feasibility of unilateral or bilateral nerve-sparing radical hysterectomy in patients with cervical cancer and evaluation of the post-surgery recovery of the bladder and rectal function. *Chinese Journal of Oncology*.

[23] Eicke M., Hohenfellner R., Leissner J. (2005). Nerve injuries in urologic surgery. *Advanced Urologic Surgery*.

[24] Zochodne D. W. (2002). Nerve and ganglion blood flow in diabetes: an appraisal. *International Review of Neurobiology*.

[25] Ralph G., Tamussino K., Lichtenegger W. (1988). Urodynamics following radical abdominal hysterectomy for cervical cancer. *Archives of Gynecology and Obstetrics*.

[26] Kadar N., Saliba N., Nelson J. H. (1983). The frequency, causes and prevention of severe urinary dysfunction after radical hysterectomy. *British Journal of Obstetrics and Gynaecology*.

[27] Forney J. P. (1980). The effect of radical hysterectomy on bladder physiology. *American Journal of Obstetrics and Gynecology*.

[28] Scotti R. J., Bergman A., Bhatia N. N., Ostergard D. R. (1986). Urodynamic changes in urethrovesical function after radical hysterectomy. *Obstetrics and Gynecology*.

[29] Low J. A., Mauger G. M., Carmichael J. A. (1981). The effect of Wertheim hysterectomy upon bladder and urethral function. *American Journal of Obstetrics and Gynecology*.

[30] Ralph G., Winter R., Michelitsch L., Tamussino K. (1991). Radicality of parametrial resection and dysfunction of the lower urinary tract after radical hysterectomy. *European Journal of Gynaecological Oncology*.

[31] Charoenkwan K., Pranpanas S. (2007). Prevalence and characteristics of late postoperative voiding dysfunction in early-stage cervical cancer patients treated with radical hysterectomy. *Asian Pacific Journal of Cancer Prevention*.

[32] Naik R., Nwabinelli J., Mayne C., Nordin A., De Barros Lopes A., Monaghan J. M., Hilton P. (2001). Prevalence and management of (non-fistulous) urinary incontinence in women following radical hysterectomy for early stage cervical cancer. *European Journal of Gynaecological Oncology*.

[33] Jackson K. S., Naik R. (2006). Pelvic floor dysfunction and radical hysterectomy. *International Journal of Gynecological Cancer*.

[34] Lin L.-Y., Wu J.-H., Yang C.-W., Sheu B.-C., Lin H.-H. (2004). Impact of radical hysterectomy for cervical cancer on urodynamic findings. *International Urogynecology Journal and Pelvic Floor Dysfunction*.

[35] Chuang T.-Y., Yu K.-J., Penn I.-W., Chang Y.-C., Lin P.-H., Tsai Y.-A. (2003). Neurourological changes before and after radical hysterectomy in patients with cervical cancer. *Acta Obstetricia et Gynecologica Scandinavica*.

[36] Oda Y., Todo Y., Hanley S., Hosaka M., Takeda M., Watari H., Kaneuchi M., Kudo M., Sakuragi N. (2011). Risk factors for persistent low bladder compliance after radical hysterectomy. *International Journal of Gynecological Cancer*.

[37] Hazewinkel M. H., Sprangers M. A. G., van der Velden J., van der Vaart C. H., Stalpers L. J. A., Burger M. P. M., Roovers J. P. W. R. (2010). Long-term cervical cancer survivors suffer from pelvic floor symptoms: a cross-sectional matched cohort study. *Gynecologic Oncology*.

[38] Butler-Manuel S., Buttery L., AHern R., Polak J., Barton D. (2000). Pelvic nerve plexus trauma at radical hysterectomy and simple hysterectomy: the nerve content of the uterine supporting ligaments. *Cancer*.

[39] Butler-Manuel S. A., Buttery L. D. K., A'Hern R. P., Polak J. M., Barton D. P. J. (2002). Pelvic nerve plexus trauma at radical and simple hysterectomy: a quantitative study of nerve types in the uterine supporting ligaments. *Journal of the Society for Gynecologic Investigation*.

[40] Maneschi F., Ianiri P., Sarno M., Gagliardi F., Panici P. B. (2012). Nerve-sparing class III-IV radical hysterectomy: urodynamic study and surgical technique. *International Journal of Gynecological Cancer*.

[41] Raspagliesi F., Ditto A., Hanozet F., Martinelli F., Solima E., Zanaboni F., Kusamura S., Fontanelli R. (2007). Nerve-sparing radical hysterectomy in cervical cancer: evolution of concepts. *Gynecologic Oncology*.

[42] Fraser A. C. (1966). The late effects of Wertheim's hysterectomy on the urinary tract. *The Journal of Obstetrics and Gynaecology of the British Commonwealth*.

[43] Seski J. C., Diokno A. C. (1977). Bladder dysfunction after radical abdominal hysterectomy. *American Journal of Obstetrics & Gynecology*.

[44] Glahn B. E. (1970). The neurogenic factor in vesical dysfunction following radical hysterectomy for carcinoma of the cervix.. *Scandinavian Journal of Urology and Nephrology*.

[45] Barclay D. L., Roman-Lopez J. J. (1975). Bladder dysfunction after Schauta hysterectomy. One year follow up. *The American Journal of Obstetrics and Gynecology*.

[46] Forney J. P. (1980). The effect of radical hysterectomy on bladder physiology. *American Journal of Obstetrics & Gynecology*.

[47] Low J. A., Mauger G. M., Carmichael J. A. (1981). The effect of Wertheim hysterectomy upon bladder and urethral function. *The American Journal of Obstetrics and Gynecology*.

[48] Griffiths D., Höfner K., van Mastrigt R., Rollema H. J., Spångberg A., Gleason D. (1997). Standardization of terminology of lower urinary tract function: pressure-flow studies of voiding, urethral resistance, and urethral obstruction. *Neurourology and Urodynamics*.

[49] Hamada K., Kihana T., Kataoka M., Yoshioka S., Nishio S., Matsuura S., Ito M. (1999). Urinary disturbance after therapy for cervical cancer: urodynamic evaluation and *β*2-agonist medication. *International Urogynecology Journal and Pelvic Floor Dysfunction*.

[50] Maman K., Aballea S., Nazir J. (2013). Comparative efficacy and safety of medical treatments for the management of overactive bladder: a systematic literature review and mixed treatment comparison. *European Urology*.

[51] Axelsen S. M., Petersen L. K. (2006). Urogynaecological dysfunction after radical hysterectomy. *European Journal of Surgical Oncology*.

[52] Haferkamp A., Dörsam J., Resnick N. M., Yalla S. V., Elbadawi A. (2003). Structural basis of neurogenic bladder dysfunction III. Intrinsic detrusor innervation. *The Journal of Urology*.

[53] Pieterse Q. D., Maas C. P., Ter Kuile M. M., Lowik M., Van Eijkeren M. A., Trimbos J. B. M. Z., Kenter G. G. (2006). An observational longitudinal study to evaluate miction, defecation, and sexual function after radical hysterectomy with pelvic lymphadenectomy for early-stage cervical cancer. *International Journal of Gynecological Cancer*.

[54] Hazewinkel M. H., Gietelink L., van der Velden J., Burger M. P. M., Stoker J., Roovers J.-P. W. R. (2012). Renal ultrasound to detect hydronephrosis: a need for routine imaging after radical hysterectomy?. *Gynecologic Oncology*.

